# Cardiac Embolism Following Delivery

**Published:** 1878-11

**Authors:** John L. Sullivan

**Affiliations:** Malden


### CARDIAC EMBOLISM FOLLOWING DELIVERY.

By JOHN L. SULLIVAN, M. D., Malden.

Mrs. C. F. B., American, in good circumstances, was delivered of her first child, a vigorous female, weighing eight pounds, on the evening of March 6th. Previous to her confinement I saw the lady once, when I gathered the following particulars: Parents both living and in good health. Her own health during childhood and before marriage usually good. When quite young she had typhoid fever, a mild form, from which she recovered without drawback. Several years ago, after violent mental emotion, was attacked with distressing palpitation, which soon ceased, and which did not prevent her attending a ball given on the same night, and dancing every "set" without inconvenience. Since then no cardiac symptoms of which she was aware, and none now discoverable by auscultation. At times troubled with leucorrhœa. During gestation health seemed to improve, "pregnancy agreeing with her;" had experienced, however, especially during the latter months, a sensation of "inward heat," as she described it, which enabled her to endure without discomfort a lower temperature than was agreeable to other persons, and to dispense with warming the rooms which she occupied.

The labor was in all respects natural, and easier than falls to the lot of most women with the first child. Head presentation, right occipito-anterior. Duration, from the earliest indication of uterine action to the expulson of the placenta, ten hours. A few moments after the birth of the child, the after-birth, expedited by external pressure, came away; the uterus contracted immediately to the typical cricket-ball size and "feel," so gratifying to the accoucheur; and when, half an hour later, I retired, both mother and child seemed "as well as could be expected."[2]

March 7th. This morning Mrs. B. was reported by the nurse to have "flowed more than usual during the night," a symptom which could not be attributed to relaxation of the womb, as that organ was found, on examination, in the same state of firm contraction it had assumed the previous night. Opium and ergot were ordered in moderate doses every third hour.

March 8th. This forenoon, on attempting to sit up in bed to pass water, the patient voided a considerable quantity of dark coagular, which had accumulated in the vagina, and for a few moments afterwards was faint. On my arrival, however, I found her quite recovered, that is, in good spirits, neither blanched in countenance nor presenting any symptoms of exhaustion. There was no subsequent hæmorrhage, although the opium and ergot were omitted, and the lochia henceforward continued scanty in quantity and greenish in color; in short, quite normal. The voiding of the coagula and the nurse's estimate of the amount of blood lost during the night following delivery are recorded, not as important in themselves, but as forming part of the clinical history of an unfortunate case.

March 9th. Very comfortable; pulse 80; temperature 99° F.; appetite good; bowels open; urine copious and of good color.

March 10th. I shared to-day the patient's disappointment (she was anxious to suckle her infant) not to find some evidence of activity in the breasts, which were more flaccid than yesterday. Other symptoms favorable.

March 11th. The breasts are still quite soft. This P. M. Mrs. B. complained of a disagreeable sensation suddenly felt in the inside of the left leg and thigh, which she described as "something between a numbness and a chilliness." This sensation speedily vanished, leaving behind it no trace of pain, swelling, redness, heat, or tenderness on pressure. And at no subsequent period in the progress of the case could any of these symptoms be detected, although inquiries were made, and the limb, including the groin, examined in its entire length at each visit. One hour after the above-described attack she was seized with violent and distressing palpitation resembling that experienced on the afternoon preceding the ball. Thereupon she burst into a paroxysm of hysterical weeping and sobbing. At this moment I entered the room. "Oh, doctor," she exclaimed, "I have that heart trouble again!" I was not long in ascertaining that something unusual had happened, for the heart was indeed beating with fearful rapidity, unlike anything I had ever before observed, attended with a heaving impulse in the præcordial region visible through the clothing. It was impossible to count the pulse at the wrist, or to determine its frequency by auscultation. It could not have been, however, less than 200 per minute. Both sounds of the heart were distinctly audible, and were accompanied with a bruit or whiff which suggested the puff of a locomotive engine at full speed. The respiration was somewhat quickened (32 per minute), but not in proportion to the pulse. Moderate difficulty of breathing was complained of, but there was no perceptible lividity of the lips or face, and the expression was not in the least anxious or distressed, so that as soon as the patient's tears were dried, and she had recovered her wonted composure, she did not *look* ill. Temperature 101°. Urine drawn by the catheter and tested for albumen, etc., gave negative results.

A mixture containing tr. castor, tr. digitalis, with spirits of nitric and sulphuric ether, was directed to be taken hourly.

March 12th. This morning Mrs. B. welcomed me with a cheerful smile, and in accents equally cheerful informed me that she was much better,—a favorable impression which I was unable to corroborate, her condition having certainly not improved. On the contrary, the pulse had become weaker, and, if possible, more rapid; the temperature had risen to 103°, and the respiration to 40 per minute. Since my last visit she had assumed the left lateral posture (with the knees drawn up), from which she could not be moved on account of the increased difficulty of breathing induced by the slightest change in her position. Notwithstanding this ominous circumstance she insisted that she had been greatly relieved by her medicine, predicted a speedy recovery, and had evidently no suspicion of impending danger. And indeed her countenance was so animated and cheerful, her expression so natural, and her general appearance so strikingly in contrast with the apparent gravity of her other symptoms that it was difficult to accept the conclusion that death was inevitable, and at hand. Still, I felt it a duty to communicate to her friends my convictions that the case was one of cardiac embolism, which would prove fatal in a few days at furthest, possibly in a few hours.

March 13th. At my request Dr. Minot saw the patient to-day. After examining the case with great care, and fully recognizing its alarming features, he ventured to express a faint hope, unhappily destined to disappointment, that we might have to do not with embolism, but with hysteria simulating that condition. Agreeably to his suggestion, the digitalis was continued in larger doses, and the same nourishing and sustaining measures that had been employed from the beginning were still persevered with. Temperature 106.5°; respiration 48–52; pulse as heretofore.

March 14th. Much worse; left knee swollen and painful; one-half of right breast perfectly soft and flaccid, the other half swollen, livid, and tense; the entire left breast in the same swollen condition; tongue swollen to four times its natural size, hard, very painful, and protruded with great difficulty; deglutition well-nigh impossible; voice inaudible; orthopnœa; failing pulse; restlessness; jactitation. Death occurred at six o'clock P.M., consciousness being retained to the last moment.

An autopsy was refused.—*Boston Med. and Surg. Journal.*

